# Sensing capabilities of the sawtooth penta-SiC_2_ nanoribbon for CO_2_ and CO molecules based on variations in molecular density: insights from a DFT investigation

**DOI:** 10.1039/d5ra02502h

**Published:** 2025-06-23

**Authors:** Tran Yen Mi, Huynh My Linh, Trung-Phuc Vo, Huynh Anh Huy

**Affiliations:** a Physics Department, College of Natural Sciences, Can Tho University 3-2 Street Can Tho Vietnam; b Faculty of Physics, School of Education, Can Tho University 3-2 Street Can Tho 900000 Vietnam hahuy@ctu.edu.vn +84 0292 3830261 +84 0918 445148

## Abstract

Using Density Functional Theory (DFT) simulations, we explore the gas-sensing capabilities of the sawtooth penta-SiC_2_ nanoribbon (p-SiC_2_-SS) for CO_2_ and CO molecules under varying concentrations. Our findings reveal that CO_2_ is significantly more difficult to capture than CO. Free CO_2_ adsorption occurs only when its initial distance from the adsorbent is less than 1.80 Å, and the molecule should be positioned parallel to the adsorbent. Under these conditions, the material’s electric field bends CO_2_ to an angle of around 135°, inducing polarity and enabling adsorption. At low concentrations (one molecule per approximately 54 × 10^−6^ cm^3^), p-SiC_2_-SS selectively adsorbs CO_2_*via* strong chemisorption, with an adsorption energy of approximately −1.60 eV. When the molecular concentration triples, p-SiC_2_-SS sequentially adsorbs both CO_2_ and CO, with the adsorption energies decreasing to approximately −0.33 eV and −0.36 eV, respectively. Additionally, the electronic properties of p-SiC_2_-SS undergo distinct modifications depending on the type of adsorbed molecule. In all cases, the p orbitals of carbon and silicon atoms predominantly contribute to the energy levels near the Fermi level, with the p orbitals of carbon atoms playing a dominant role at the CBM. Our study highlights the potential of p-SiC_2_-SS as an effective gas sensor, capable of detecting and distinguishing CO_2_ and CO molecules, especially across different molecular concentrations.

## Abstract 2

The adsorption mechanism as a function of (a) total energy and (b) the angle *θ* of CO_2_*vs.* distance *d* of CO_2_@p-SiC_2_-SS.

## Graphical abstract

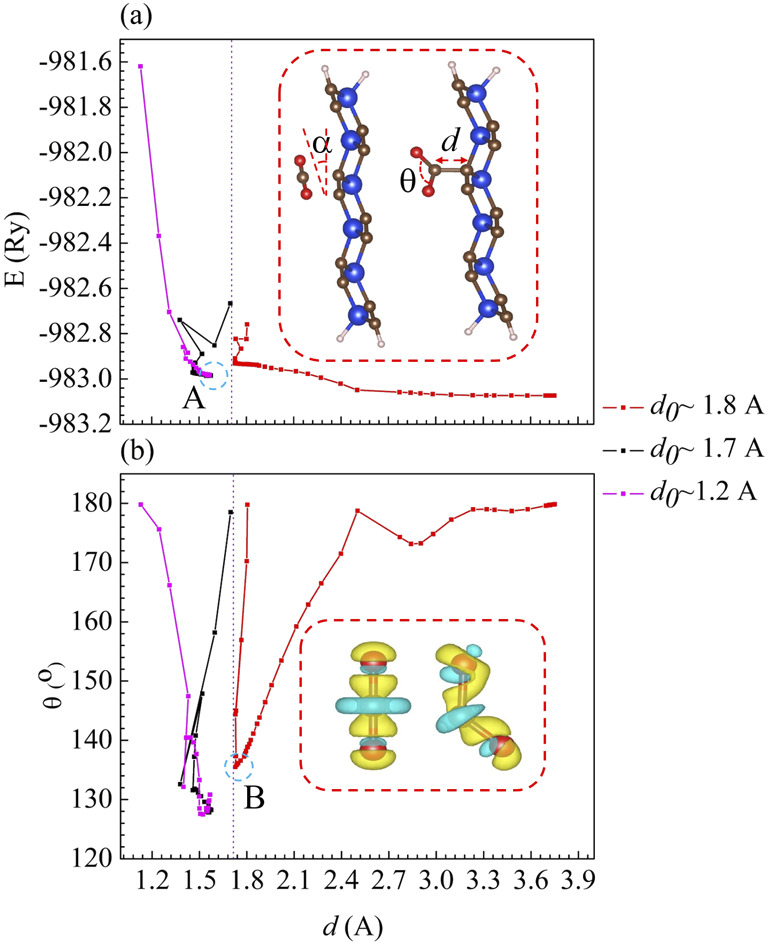

## 1 Introduction

Air pollution remains a critical challenge in our modern society, contributing to severe consequences such as ozone layer depletion, the greenhouse effect, and various health disorders.^1–5^ Consequently, the development of nanostructure-based gas sensors is of significant interest and priority in addressing these issues.^6–9^ With the support of first-principles calculations, various simulations have been performed to study nanoscale materials in terms of their gas-sensing capabilities to detect harmful gas molecules, such as CO_2_ and CO. Between 2019 and 2024, the selected adsorbents have typically been 2D structures or clusters. These studies reveal several challenges in sensing CO_2_ and CO molecules. For example, it is difficult for graphite,^10^ single layer penta-PdSe_2_ and penta-PdPSe,^11^ and penta-NiPN^12^ to capture CO_2_ and CO effectively. However, applying an appropriate external electric field^13^ to penta-graphene (PG) could makes CO_2_ adsorption easier and controllable. Additionally, doping Fe atoms on PG^14^ or metal oxides on C_60_ (ref. 15) plays a crucial role in enhancing the capture of CO_2_ and CO. Nevertheless, all of these studies have not addressed the ability of the adsorbents to distinguish between CO_2_ and CO. For host materials that are metal particles (Co_*n*_, Ni_*n*_, Cu_*n*_, Fe_*n*_),^16,17^ the particle size and the initial relative position of CO_2_ or CO are important factors in determining their interaction. Specifically, under the same conditions, CO is more readily adsorbed than CO_2_. In addition, CO can also be a transformation product of CO_2_ after the interaction between CO_2_ and a graphene cluster,^18^ or both types of molecules can be structurally broken when interacting with a BeP_2_ single layer.^19^ To enhance the sensitivity of nanoscale materials, substitutional doping into single layer models is usually employed. For instance, carbon (C) atoms in graphene^20^ and penta-graphene (PG)^21^ are commonly substituted by silicon (Si),^22^ germanium (Ge),^23^ or tin (Sn).^24^ Among these dopants, Si is a preferred choice due to its structural similarity to C. For example, p-SiC_2_ (ref. 25) is a 2D model created by replacing sp^3^-hybridized C atoms in PG with Si atoms. This structure has been predicted in experiments.^26^ Nanoribbon structures derived from these 2D materials are attracting significant interest in gas sensor research.^27,28^ Specifically, p-SiC_2_-SS is a newly proposed non-magnetic nanoribbon system^29–31^ derived from p-SiC_2_. It features sawtooth-shaped (SS) edges, where edge dangling bonds are typically neutralized by hydrogen (H) atoms. Although p-SiC_2_-SS resembles PG-SS,^28,31,32^ it exhibits significantly different electronic structures and electronic transport properties. For instance, the Valence Band Maximum (VBM) and Conduction Band Minimum (CBM) states of p-SiC_2_-SS are mainly concentrated in the central region of the structure, whereas PG-SS has more spatially separated electronic states; the electronic and electronic transport properties of p-SiC_2_-SS are influenced by both sp^2^- and sp^3^-hybridized atoms, unlike PG-SS which is dominated by sp^2^-hybridized atoms only; the energy band gap (*E*_g_) of p-SiC_2_-SS is smaller than that of PG-SS for the same ribbon width. With the highly stable penta-1D morphology, rich quantum effects, and essential properties, the p-SiC_2_-SS system demonstrates potential application prospects in gas sensing with high capacity and cost-effectiveness. This motivates us to investigate the gas-sensing characteristics of p-SiC_2_-SS for CO_2_ and CO gases, complementing our previous research on the adsorption capacity of PG-SS.^33^ The content of this article is divided into the following sections. In the Computational method section, the computation parameters and models are provided. In addition, in the Results and discussion section, for each kind of gas molecule, we first investigate the adsorption mechanism. This involves analyzing key terms related to the adsorption structure, such as adsorption energy (*E*_ad_); the molecular configuration of the gas molecule (p-SiC_2_-SS is kept fixed during the adsorption process); and the charge transfer as determined by electron density (ED) and electron density difference (EDD) analyses. These results provide insight into the nature of the interaction between the gas molecule and the adsorbent. Subsequently, to assess the sensing performance of p-SiC_2_-SS, we analyze its electronic properties, focusing on the band structure (BS), density of states (DOS), and projected density of states (PDOS). Specifically, we calculate the change in the band gap (Δ*E*_g_) and examine newly emerging energy levels near the Fermi level, along with their dispersion. Furthermore, if Δ*E*_g_ or the dispersion of these new energy levels continues to vary with different gas molecules or molecular concentration, it suggests that p-SiC_2_-SS is capable of detecting both gas species and fluctuations in their concentrations. Finally, our main findings are summarized in the Conclusions section.

## 2 Computational method

Calculations are made using the basis of density functional theory (DFT)^34^ implemented in the Quantum ESPRESSO code.^35^ The generalized gradient approximation (GGA) with the Perdew–Burke–Ernzerhof (PBE) functional^36^ is used to treat the exchange–correlation energy, using SSSP Efficiency PBE v1.3 pseudopotentials from the SSSP library^37^ for H, C, Si^38^ and for O.^39^ To obtain optimal configurations, we set a pressure of 0.5 kbar, a maximum force of 10^−3^ Ry bohr^−1^ on each atom, and a total energy convergence threshold of approximately 2.2 × 10^−4^ Ry. The Brillouin zone is sampled by a 1 × 1 × 10 grid for geometric optimization and a 1 × 1 × 15 grid for density of states (DOS) calculations. A vacuum space of 23 Å is applied perpendicularly to the adsorbent to prevent interaction between adjacent repeating structures. The van der Waals interaction^40^ is included to account for interactions between the molecule and the material. Vesta software^41^ is utilized to visualize the models and their electron density distributions. A unit cell of p-SiC_2_-SS, with a width of six sawtooth chains, is first constructed and optimized. A three-unit cell of p-SiC_2_-SS, referred to as the p-SiC_2_-SS supercell, is then built to serve as the adsorbent with fixed atomic positions. Subsequently, we systematically investigate the adsorption between the adsorbent and each type of gas molecule at different concentrations. The adsorption models of one CO_2_, three CO_2_, one CO, and three CO molecules on the p-SiC_2_-SS supercell are referred to as CO_2_@p-SiC_2_-SS, 3CO_2_@p-SiC_2_-SS, CO@p-SiC_2_-SS and 3CO@p-SiC_2_-SS, respectively. The formula used to calculate adsorption energy has the following form:

1 *E*_ad_ = *E*_tot_ − (*E*_sub_ + *E*_mole_)

where *E*_ad_ and *E*_tot_ are the adsorption energy and total energy of the whole system, respectively; *E*_sub_ and *E*_mole_ are the energy of the host material and isolated molecule, respectively.

## 3 Results and discussion

### 3.1 Electronic structure of p-SiC_2_-SS

**Fig. 1:**
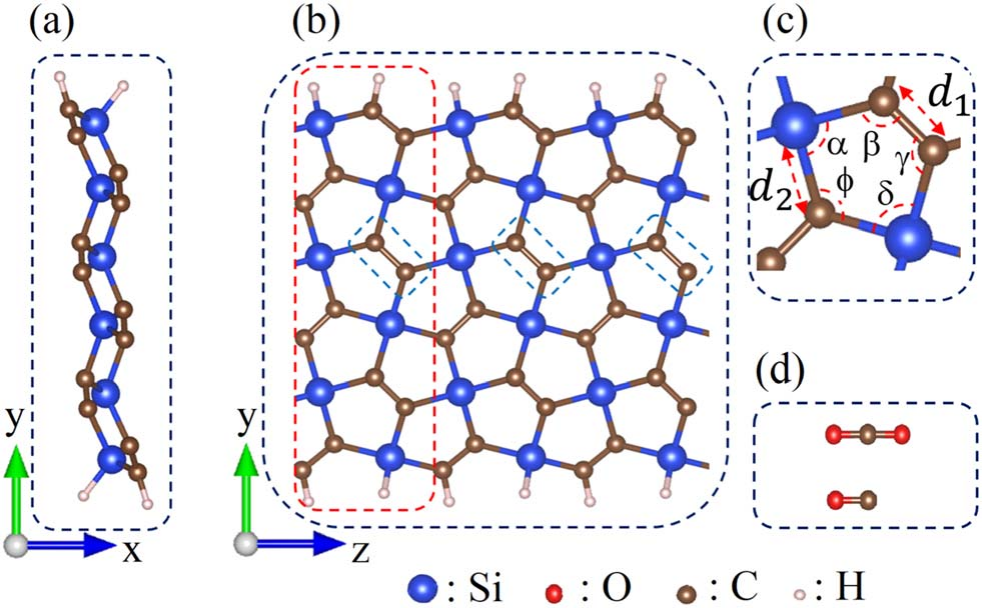
Optimized structure of p-SiC_2_-SS supercell. (a) Side and (b) top views. The dashed red rectangle indicates the unit cell of the ribbon, while the dashed blue ones mark the initial adsorption sites for CO_2_ and CO in turn. (c) Geometric structure of a representative pentagonal ring. (d) Isolated CO_2_ and CO molecules. The blue, red, brown and white spheres represent Si, O, C and H atoms, respectively.

The adsorbent is formed by tripling the optimized p-SiC_2_-SS unit cell. Fig. 1 represents the optimized geometric structure of the p-SiC_2_-SS supercell from the side (Fig. 1a) and the top (Fig. 1b). In particular, the red dashed rectangle in Fig. 1b highlights the optimized p-SiC_2_-SS unit cell, while the blue dashed rectangles mark the initial molecular adsorption sites. If the system adsorbs CO_2_ or CO (Fig. 1d), that molecule is placed in one of these three relative positions. If the molecular concentration triples, three molecules are placed simultaneously in these three relative positions. In addition, the structural parameters of a typical pentagonal ring in the nanoribbon are illustrated in Fig. 1c, with *d*_1_ ≡ C–C = 1.36 Å, *d*_2_ ≡ C–Si = 1.90 Å, *α* ≡ C–Si–C = 96.90°, *β* ≡ Si–C–C = 117.78°, *γ* ≡ C–C–Si = 117.00°, *δ* ≡ C–Si–C = 97.12° and *φ* ≡ Si–C–Si = 109.56°. These values are consistent with previous research.^29,30^

**Fig. 2:**
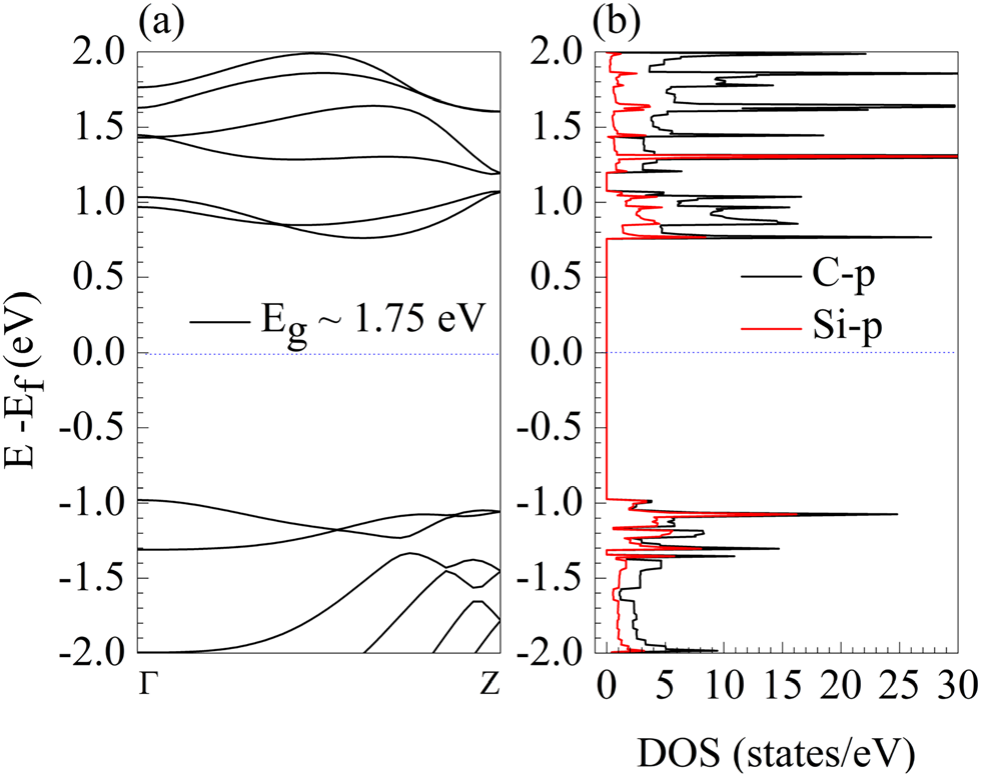
Typical electronic properties of the p-SiC_2_-SS unit cell. (a) Band structure (BS), and (b) density of states (DOS). The dashed blue lines indicate the Fermi level set as the reference energy in each diagram.

Moreover, the exact electronic properties of the p-SiC_2_-SS supercell are confirmed by BS and DOS calculations on the p-SiC_2_-SS unit cell, as shown in Fig. 2. These results confirm that the adsorbent is a semiconductor with an indirect band gap of approximately *E*_g_ = 1.75 eV. Additionally, its electronic properties are mainly governed by the p orbitals of C (C-p) and Si (Si-p). These values are in good agreement with previous research.^29,31^

### 3.2 CO_2_ adsorption

**Fig. 3:**
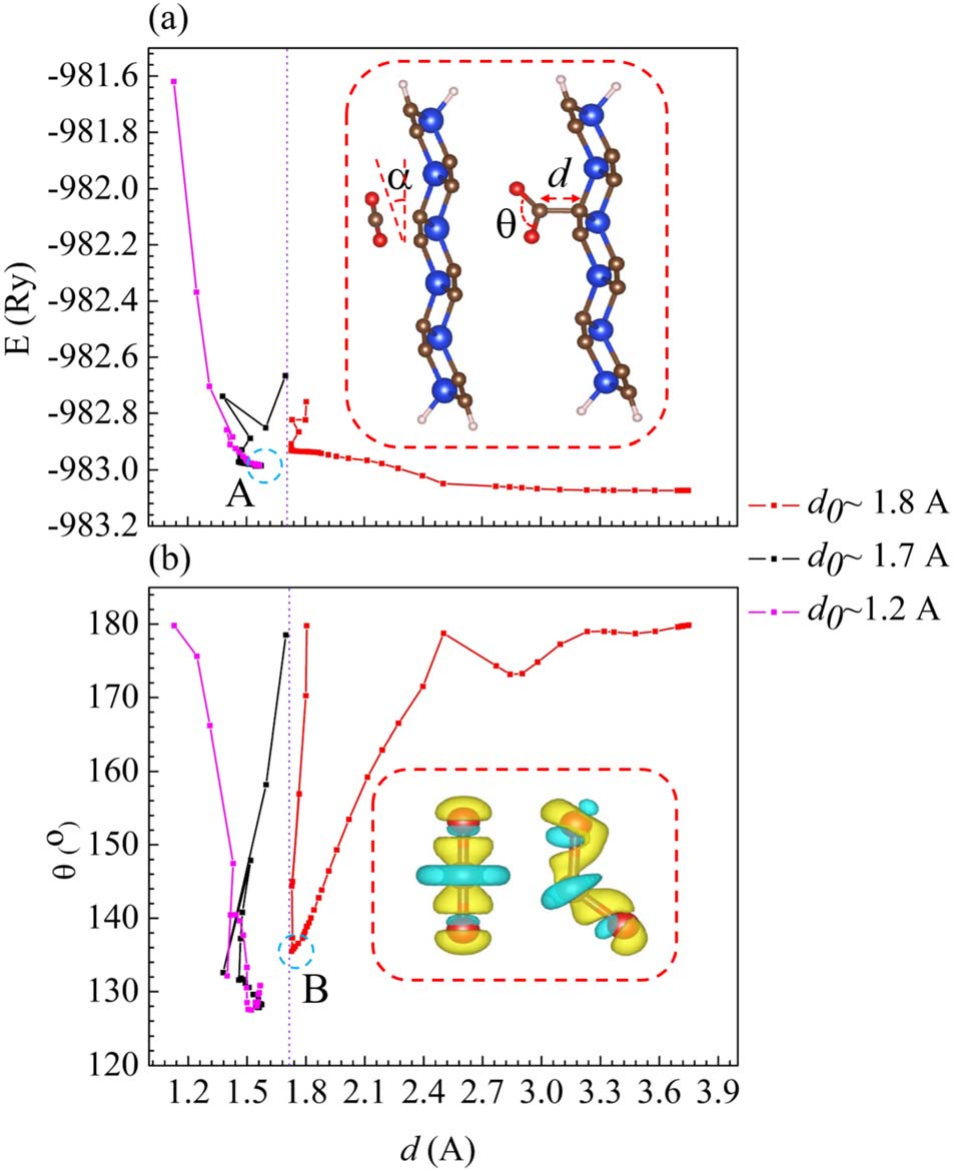
The adsorption mechanism as a function of (a) total energy and (b) the angle *θ* of CO_2_*vs.* distance *d* of CO_2_@p-SiC_2_-SS. Dashed circle A in (a) indicates the optimized structure. The inset in (a) depicts the angle *α* between free CO_2_ and adsorbent, along with the instantaneous *θ* and distance *d*. Dashed circle B in (b) represents the critical angle *θ* for the adsorption. The inset in (b) illustrates the variation in the electron density difference (EDD) of CO_2_ at *θ* = 180° and 135°, where yellow and cyan regions indicate areas of electron gain and loss, respectively.

In the first stage of adsorption, the free CO_2_ is placed parallel (*α* = 0°) and directly above the C–C junction of p-SiC_2_-SS, with its initial distance denoted as *d*_o_. Here, *d*_o_ = C_CO_2__–C_sub_ represents the separation between the C atom of CO_2_ and the nearest C–C junction, while its instantaneous value during the adsorption process is *d* (the inset in Fig. 3a). When *d*_o_ is set to 1.80 Å, 1.70 Å, and 1.20 Å, three corresponding adsorption processes (marked in red, black, and purple) are observed, as shown in Fig. 3. We monitor the optimization process by analyzing variations in the total energy of CO_2_@p-SiC_2_-SS (Fig. 3a) and the angle *θ* ≡ O–C–O of CO_2_ (Fig. 3b) as a function of *d*. From Fig. 3a, we observe that if *d*_o_ is less than 1.80 Å, a chemical bond is likely to form, as indicated by dashed circle A. At this point, *θ* = 128.17° and distance *d* = 1.54 Å.^42^ Since CO_2_ is a nonpolar molecule in its free state, the interaction first causes it to bend into a polar structure. Fig. 3b shows that when *θ* < 135°, marked by dashed circle B, the polarization of CO_2_ becomes sufficient to form a strong bond with p-SiC_2_-SS. The inset in Fig. 3b illustrates the EDD of CO_2_ as *θ* changes from 180° (nonpolar structure) to 135° (polar structure), with yellow and cyan regions representing electron gain and loss, respectively.

The influence of the angle *α* on CO_2_@p-SiC_2_-SS is systematically investigated through several representative cases, as summarized in Table 1. Cases 1, 2, and 3 demonstrate that negligible interaction occurs (*E*_ad_ = −0.03 eV) for all *α* values when *d*_0_ = 1.80 Å. These results indicate that changing *α* does not affect the adsorption mechanism when the initial separation is around 1.80 Å. Case 5 reveals that even at a shorter initial distance of *d*_0_ = 1.51 Å, the interaction remains weak (*E*_d_ = −0.03 eV) when *α* = 45°. This result shows that even with *d*_0_ < 1.80 Å, a nonzero *α* still prevents p-SiC_2_-SS from capturing CO_2_. Notably, based on the optimized structures of cases 4 and 6, it is clear that a strong chemical interaction occurs in CO_2_@p-SiC_2_-SS, forming a single stable configuration as confirmed in Fig. 3. All cases from Table 1 confirm that in addition to requiring an initial distance less than 1.80 Å, the initial angle *α* must be 0° for a strong chemical bond to form in CO_2_@p-SiC_2_-SS. Examining the adsorption energies (*E*_ad_ ≈ +1.16 eV for cases 4 and 6, calculated using eqn (1)), these values seem to contradict the corresponding optimized adsorption structures. However, when we replace *E*_mol_ in eqn (1) with the energy of CO_2_ in a bent configuration at *θ* ≈ 135°, the adsorption energies adjust to *E*_ad_ = −1.60 eV. This suggests that, with an initial distance *d*_0_ < 1.80 Å and *α* = 0°, the electric field of p-SiC_2_-SS first bends and polarizes CO_2_. Then it is gradually pulled toward the adsorbent as its bending angle approaches *θ* ≈ 135°. Finally, a chemical bond is formed when *d* = 1.54 Å and *θ* = 128.26°. Therefore, all cases in Table 1 not only confirm the critical role of *α* in the adsorption mechanism of CO_2_@p-SiC_2_-SS, but also reveal how p-SiC_2_-SS captures CO_2_.

**Table 1:** The influence of *α* and initial distance *d*_0_ for the optimized configuration of CO_2_@p-SiC_2_-SS

No.	Initial structure	Optimized structure	*E*_ad_ (eV)
1	*d*_0_ = 1.80 Å	*d* = 3.72 Å	−0.03
	*α* = 0°	*θ* = 179.78°	
2	*d*_0_ = 1.80 Å	*d* = 3.23 Å	−0.03
	*α* = 90°	*θ* = 179.69°	
3	*d*_0_ = 1.80 Å	*d* = 3.22 Å	−0.01
	*α* = 45°	*θ* = 178.40°	
4	*d*_0_ = 1.70 Å	*d* = 1.54 Å	+1.16
	*α* = 0°	*θ* = 128.26°	
5	*d*_0_ = 1.51 Å	*d* = 3.65 Å	−0.03
	*α* = 45°	*θ* = 179.70°	
6	*d*_0_ = 1.20 Å	*d* = 1.55 Å	+1.17
	*α* = 0°	*θ* = 128.17°	

**Fig. 4:**
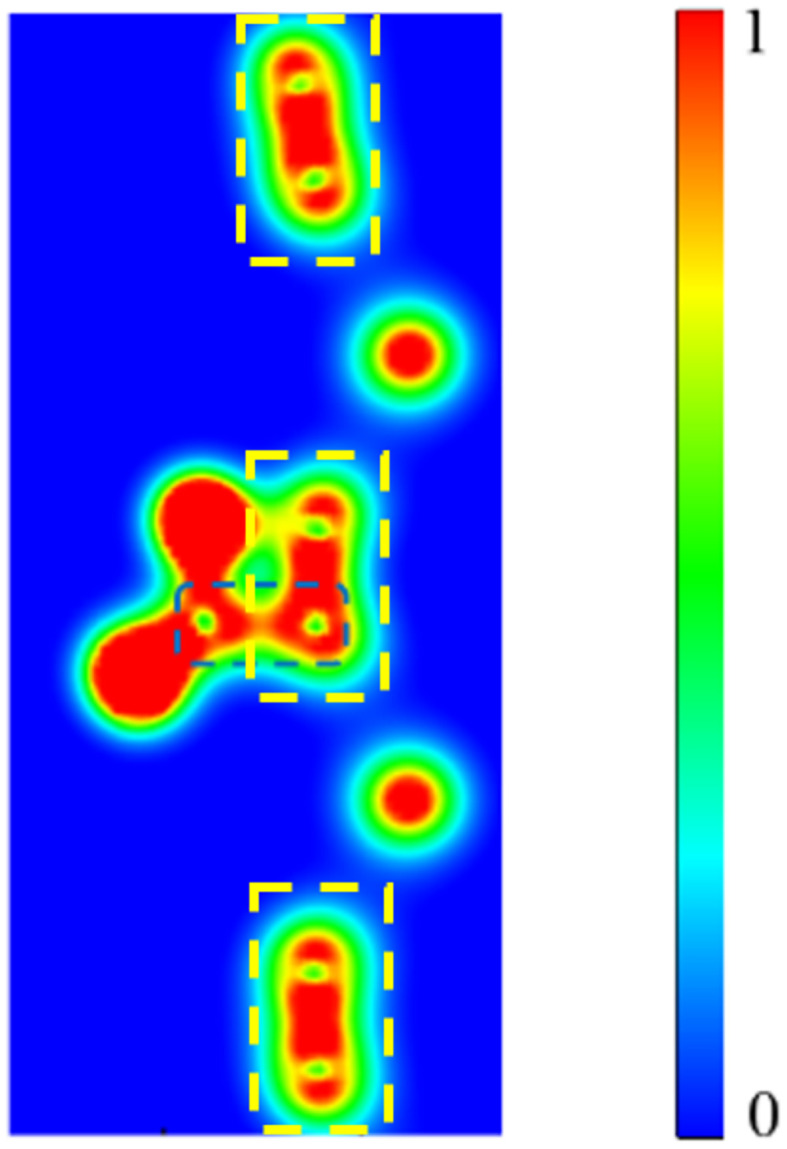
Electron density (ED) distribution between CO_2_ and p-SiC_2_-SS in a special cross-sectional view. The dashed blue rectangle highlights the electron presence at the C_CO_2__–C_sub_ bond, indicating a strong interaction. The dashed yellow rectangles show the electron distribution at the C–C junctions of the adsorbent. The color bar represents electron density variation, ranging from the lowest values (blue) to the highest values (red).

To illustrate chemisorption in CO_2_@p-SiC_2_-SS, we analyze the electron density (ED) distribution in Fig. 4. This cross-sectional view is chosen to highlight the bonding between CO_2_ and p-SiC_2_-SS with ED increasing from blue to red. The primary interaction is enclosed within the dashed blue rectangle, where the C_CO_2__–C_sub_ bond forms, with the intense red color in this region confirming a strong chemical interaction. A comparison of the electron density (ED) at the C–C bond nearest to CO_2_ with other C–C bonds in p-SiC_2_-SS—all highlighted by dashed yellow rectangles—reveals a significant difference in ED between the bond that has interacted with CO_2_ and the remaining C–C ones.

**Fig. 5:**
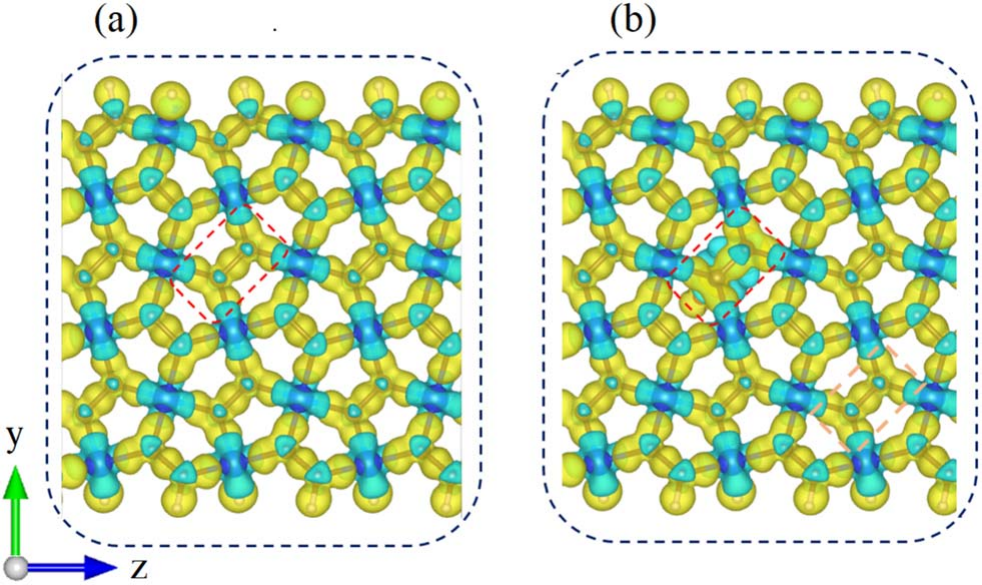
The electron density difference (EDD) of p-SiC_2_-SS in (a) the pristine case and (b) CO_2_@p-SiC_2_-SS. The dashed red and orange rectangles stand for specific C–C junctions of interest, where yellow and cyan regions indicate areas of electron gain and loss, respectively.

Fig. 5 provides a detailed illustration of how CO_2_ affects the electron density distribution at the C–C junction to which it is directly bonded, as analyzed through the EDD. In these images, yellow regions indicate electron accumulation, while blue regions represent electron depletion. The dashed red rectangles highlight the same C–C before (Fig. 5a) and after (Fig. 5b) bonding with CO_2_. It is evident that one C gains more electrons (as indicated by the expanded yellow region around it) while the other C loses electrons (as seen from the enlarged blue region) due to CO_2_ adsorption. Furthermore, when comparing the C–C inside the red rectangle in Fig. 5a with another one inside the orange rectangle in Fig. 5b—one that is not directly connected to CO_2_—we observe that their EDDs are almost the same. This further confirms that CO_2_ primarily affects the C atoms directly bonded to it, while the electronic structure of others in the model remains largely unaltered.

**Fig. 6:**
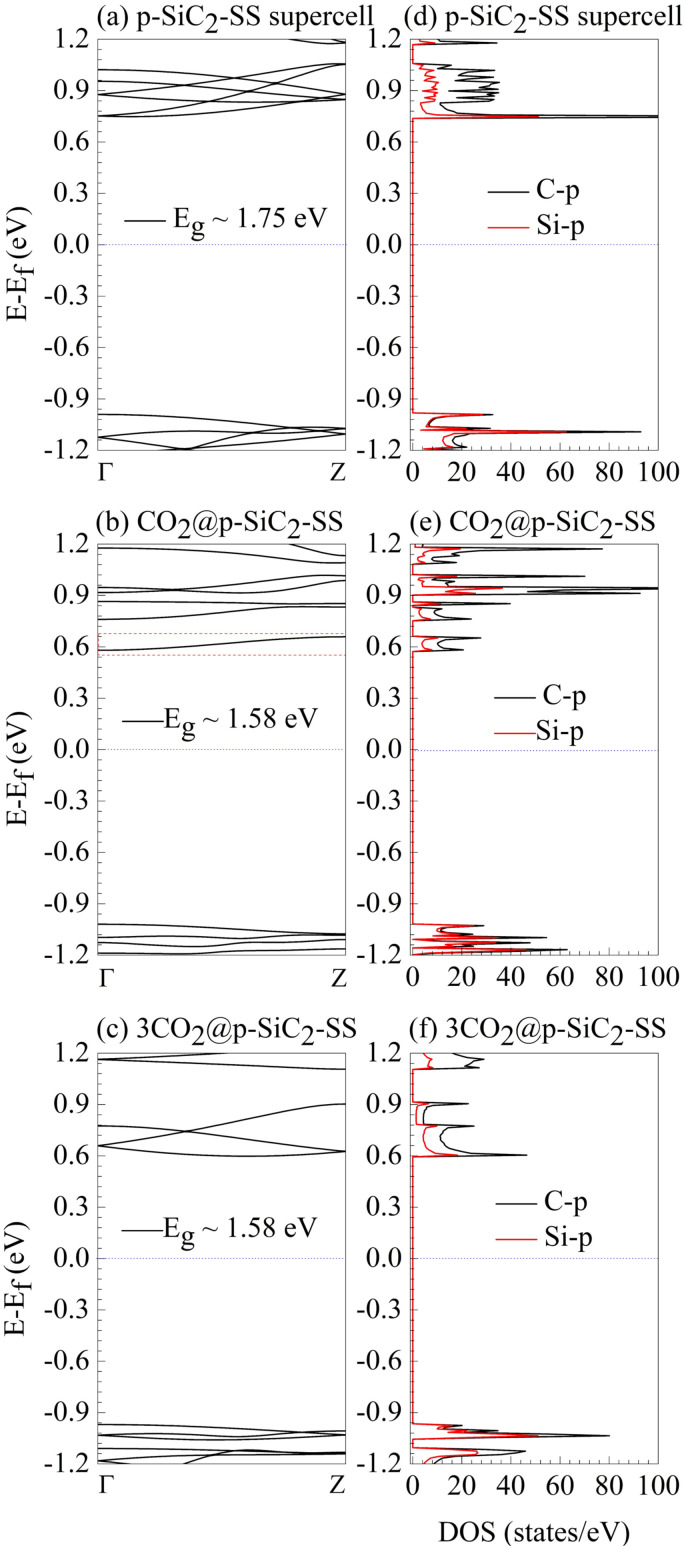
Band structure (BS) and density of states (DOS) analysis of p-SiC_2_-SS under different CO_2_ adsorption conditions: (a and d) pristine case, (b and e) CO_2_@p-SiC_2_-SS, and (c and f) 3CO_2_@p-SiC_2_-SS. The dashed red rectangle in (b) highlights new electronic states induced by CO_2_ adsorption. The dashed blue lines indicate the Fermi level set as the reference energy in each diagram.

To investigate the effect of CO_2_ concentration on the electronic properties of p-SiC_2_-SS, we compare its band structure (BS) and density of states (DOS) in three cases: the pristine system (Fig. 6a and d), CO_2_@p-SiC_2_-SS (Fig. 6b and e), and 3CO_2_@p-SiC_2_-SS (Fig. 6c and f). The BS analysis confirms that p-SiC_2_-SS remains a semiconductor with an indirect band gap, regardless of CO_2_ concentration. The DOS results further reveal that in all cases, the electronic states are primarily dictated by the C-p and Si-p orbitals, with the CBM being dominated by C-p states. Comparing Fig. 6a and d with Fig. 6b and e, we observe that CO_2_ introduces a new electronic state just above the Fermi level, highlighted by the dashed red rectangle in Fig. 6b. This new state, primarily contributed by C-p and Si-p orbitals, is responsible for the band gap reduction from 1.75 eV to 1.58 eV. Notably, when increasing the CO_2_ adsorption density to three molecules, new additional states appear right above the Fermi level also, but no further band gap reduction occurs. Instead, a distinctive feature emerges: highly dispersive energy levels appear in these new states of 3CO_2_@p-SiC_2_-SS (Fig. 6c and f), compared to those in CO_2_@p-SiC_2_-SS (Fig. 6b and e), suggesting enhanced electronic transport properties due to the increased molecular concentration. Structurally, increasing the CO_2_ concentration does not significantly alter the optimal adsorption configuration. The bond length *d* remains ∼1.56 Å, and the CO_2_ bending angle *θ* is ∼128.86°, consistent with CO_2_@p-SiC_2_-SS. However, the adsorption energy (*E*_ad_) drops drastically to −0.33 eV, which is 4.85 times lower than that of CO_2_@p-SiC_2_-SS. This significant reduction suggests that intermolecular CO_2_ interactions weaken their binding to p-SiC_2_-SS, potentially enhancing adsorbent recovery. In summary, while increasing CO_2_ concentration does not further reduce the band gap compared to CO_2_@p-SiC_2_-SS, it induces new, highly dispersive electronic states right above the Fermi level, highlighting the potential of p-SiC_2_-SS for CO_2_ detection across varying concentrations. Moreover, the decreasing adsorption energy at higher CO_2_ densities suggests an improved regeneration of adsorbent after CO_2_ capture.

### 3.3 CO adsorption

**Fig. 7:**
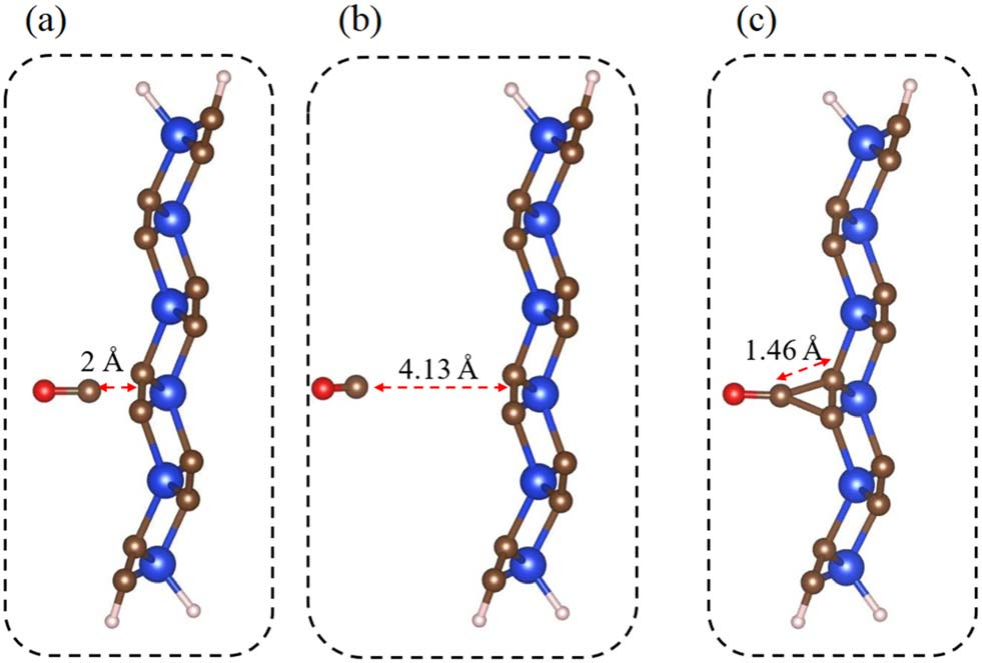
Configurations between CO and the p-SiC_2_-SS in (a) initial set-up, (b) CO@p-SiC_2_-SS and (c) 3CO@p-SiC_2_-SS.

Similarly, we examine the interactions between CO molecules and the p-SiC_2_-SS supercell at different molecular densities. Our findings indicate that CO molecular density significantly influences their connection, as shown in Fig. 7. Based on previous studies,^29,33^ CO is initially positioned directly above the C–C junction in the central region of the ribbon, with a separation of approximately 2.0 Å (Fig. 7a). For CO@p-SiC_2_-SS, there is minimal interaction between them, as evidenced by the large equilibrium distance *d* = 4.13 Å (Fig. 7b) and the weak adsorption energy *E*_ad_ = −0.12 eV. However, when the CO adsorption density is tripled, this interaction strengthens significantly. The equilibrium distance decreases to *d* = 1.46 Å (Fig. 7c), which is shorter than the average C–C bond length (∼1.54 Å),^42^ and the corresponding *E*_ad_ increases to −0.36 eV. These results suggest that increasing the CO molecular concentration enhances the adsorption efficiency of p-SiC_2_-SS for these molecules. We attribute this enhanced adsorption to CO–CO interactions, which promote stronger binding between CO molecules and p-SiC_2_-SS.

**Fig. 8:**
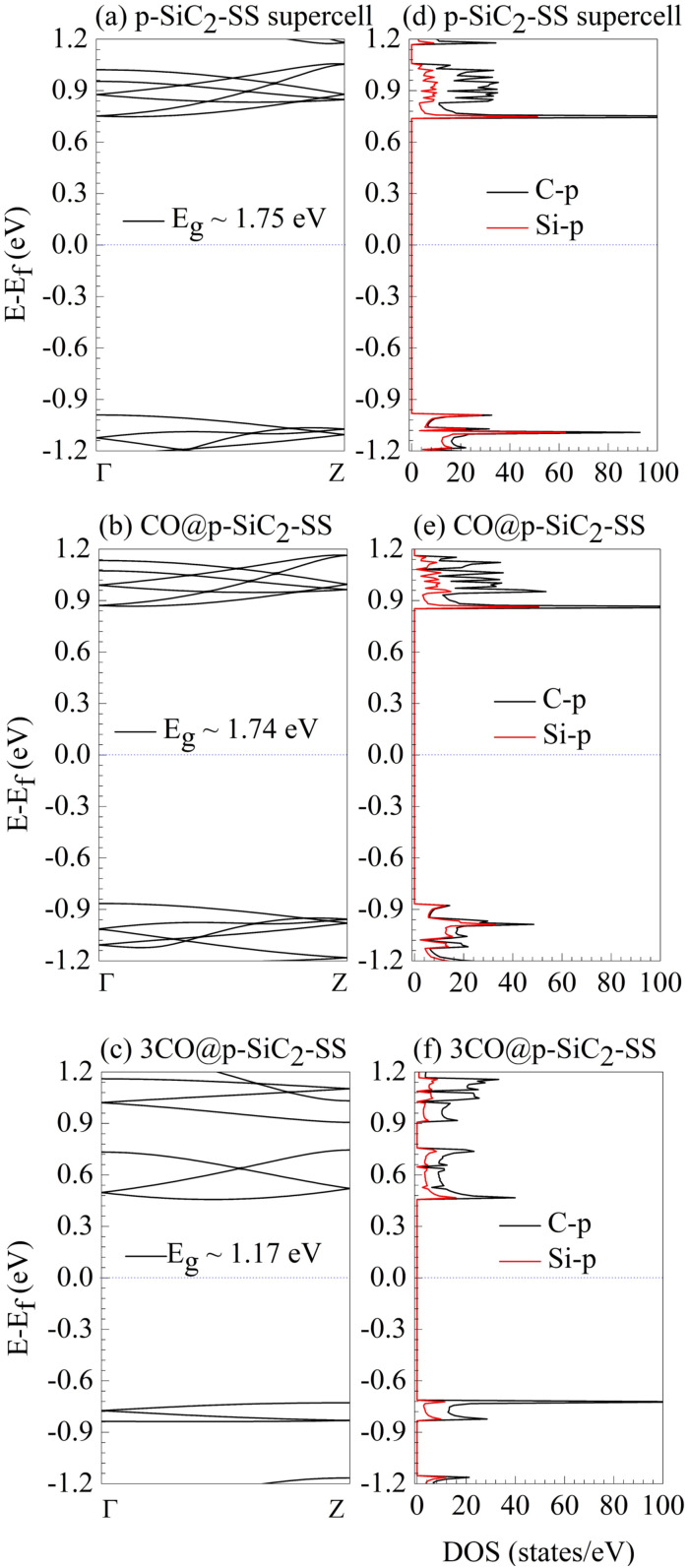
Band structure (BS) and density of states (DOS) analysis of p-SiC_2_-SS under different CO adsorption conditions: (a and d) pristine case, (b and e) CO@p-SiC_2_-SS, and (c and f) 3CO@p-SiC_2_-SS. The dashed blue lines indicate the Fermi level set as the reference energy in each diagram.

The sensing capability of p-SiC_2_-SS under varying CO concentrations is analyzed *via* BS and DOS, as shown in Fig. 8. We compare its electronic properties in three cases: the pristine system (Fig. 8a and d), CO@p-SiC_2_-SS (Fig. 8b and e), and 3CO@p-SiC_2_-SS (Fig. 8c and f). Regardless of CO density, p-SiC_2_-SS remains a semiconductor with an indirect band gap. Its electronic properties are primarily governed by C-p and Si-p orbitals, with C-p ones playing a dominant role at the CBM. In the case of CO@p-SiC_2_-SS (Fig. 8b and e), the band gap (*E*_g_) and electronic states near the Fermi level remain almost unchanged compared to the pristine case (Fig. 8a and d), suggesting that p-SiC_2_-SS is ineffective for CO detection at low concentrations (one molecule in 54 × 10^−6^ cm^3^). This result is in stark contrast to CO_2_@p-SiC_2_-SS, reinforcing our belief that p-SiC_2_-SS can effectively distinguish between CO and CO_2_ at low density. However, when the CO concentration is tripled, the electronic properties of p-SiC_2_-SS undergo a significant transformation. A comparison between the BS and DOS of 3CO@p-SiC_2_-SS (Fig. 8c and f) and the pristine case (Fig. 8a and d) reveals a notable band gap reduction from 1.75 eV to 1.17 eV, which contrasts sharply with 3CO_2_@p-SiC_2_-SS in Fig. 6. Moreover, the energy levels above the Fermi level become more dispersive, and those below the Fermi level exhibit distinct separation. These results indicate that increased CO concentration strongly affects the electronic structure of p-SiC_2_-SS. Notably, the emergence of a high-density state (115 states per eV) within the energy range of −0.71 eV to −0.84 eV (Fig. 8f) suggests the possible existence of leakage current, a feature absent in 3CO_2_@p-SiC_2_-SS (Fig. 6f). This reinforces our hypothesis that the sensing by p-SiC_2_-SS of CO and CO_2_ at high molecular density (three molecules in 54 × 10^−6^ cm^3^) is also fundamentally different.

## 4 Conclusions

In this study, we employ density functional theory (DFT) as implemented in the Quantum ESPRESSO (QE) software to investigate the sensing capabilities of the sawtooth penta-SiC_2_ nanoribbon (p-SiC_2_-SS) for CO_2_ and CO molecules under varying concentrations. Our findings indicate that CO_2_ is a challenging molecule to capture. p-SiC_2_-SS can only adsorb it if their initial configuration meets a specific condition: the free CO_2_ molecule must be oriented parallel to the adsorbent at a distance of less than 1.80 Å. Then, the electric field of the adsorbent bends CO_2_ forming an angle of less than 135°, which induces polarity in CO_2_, thereby initiating the adsorption process. At low concentration (one molecule per approximately 54 × 10^−6^ cm^3^), p-SiC_2_-SS exclusively captures CO_2_, with an adsorption energy of around −1.60 eV, while effectively detecting the molecule. This behavior distinguishes p-SiC_2_-SS from PG-SS in a remarkable way. When the molecular concentration triples, the model captures both CO_2_ and CO molecules *via* weaker chemisorption with moderate adsorption energies of approximately −0.33 eV, thereby enhancing the material’s recoverability; the electronic properties of p-SiC_2_-SS also undergo noticeable changes in both cases. Notably, in the case of 3CO@p-SiC_2_-SS, a significant increase in the density of states at the VBM suggests the potential occurrence of leakage current—an effect not observed as prominently in 3CO_2_@p-SiC_2_-SS. Compared with previous results listed in the Introduction section regarding CO_2_ and CO sensitivity, we found that p-SiC_2_-SS not only significantly distinguishes between CO_2_ and CO—particularly at high molecular densities—but also does not convert CO_2_ into CO, unlike graphene clusters or the BeP_2_ monolayer. This characteristic contributes to environmental safety. Based on these results, we reveal that p-SiC_2_-SS offers several outstanding advantages in adsorbing CO_2_ and CO under varying molecular concentrations, making it a strong candidate for gas sensor development. Moreover, due to these unique characteristics of p-SiC_2_-SS, our further research will focus on its quantitative sensing capabilities for environmentally hazardous gas molecules, in which the further optimization/tuning of the system to enhance its sensing performance will be considered, including dopings and vacancies.

## Conflicts of interest

There are no conflicts of interest to declare.

## Data Availability

The data that support the findings of this study are available from the corresponding author upon reasonable request.
